# Application of median lethal concentration (LC_50_) of pathogenic microorganisms and their antigens in vaccine development

**DOI:** 10.1186/s13104-020-05126-x

**Published:** 2020-06-15

**Authors:** Saganuwan Alhaji Saganuwan

**Affiliations:** Department of Veterinary Pharmacology and Toxicology, College of Veterinary Medicine, Federal University of Agriculture, P.M.B. 2373, Makurdi, Benue Nigeria

**Keywords:** Vaccine, Pathogenicity, Model, Arithmetic, Development, Colony forming unit

## Abstract

**Objective:**

Lack of ideal mathematical models to qualify and quantify both pathogenicity, and virulence is a dreadful setback in development of new antimicrobials and vaccines against resistance pathogenic microorganisms. Hence, the modified arithmetical formula of Reed and Muench has been integrated with other formulas and used to determine bacterial colony forming unit/viral concentration, virulence and immunogenicity.

**Results:**

Microorganisms’ antigens tested are *Staphylococcus aureus, Streptococcus pneumoniae*, *Pseudomonas aeruginosa* in mice and rat, *Edwardsiella ictaluri*, *Aeromonas hydrophila*, *Aeromonas veronii* in fish, New Castle Disease virus in chicken, Sheep Pox virus, Foot-and-Mouth Disease virus and Hepatitis A virus in vitro, respectively. The LC_50_s for the pathogens using different routes of administrations are 1.93 × 10^3^(sheep poxvirus) and 1.75 × 10^10^ for *Staphylococcus aureus* (ATCC29213) in rat, respectively. Titer index (TI) equals N log_10_ LC_50_ and provides protection against lethal dose in graded fashion which translates to protection index. N is the number of vaccine dose that could neutralize the LC_50_. Hence, parasite inoculum of 10^3^ to 10^11^ may be used as basis for determination of LC_50_ and median bacterial concentrations (BC_50_).Pathogenic dose for immune stimulation should be sought at concentration about LC_10_.

## Introduction

Many countries have renewed effort towards development of vaccine against a number of infectious diseases, such as mastitis caused by *Staphylococcus aureus* in bovine and human [1]. Capsular polysaccharide, virulent antigens [2, 3] using adhesive proteins [4] as immunogenic derivatives, deoxyribonucleic acid (DNA), autolysin and protein-binding polysaccharides are also used to stimulate immune system [5–7]. However, Saganuwan reported toxicological basis of antidote [8] and a number of vaccines presently being developed is based on modified arithmetical method of Reed and Muench [9]. Hence numbers of colony forming units of some pathogenic bacteria, viruses and their antigens were determined, using median lethal concentrations (LC_50_s) established in laboratories, with intent to calculating immunogenic doses of various infectious agents.

## Main text

### Methods

Reference was made to journal articles on development of vaccines against methicillin resistance *Staphylococcus aureus* and other pathogenic microorganisms that cause diseases in human and animals. Median lethal concentrations (LC_50_s) of *Staphylococcus aureus*, *Streptococcus pneumoniae*, *Pseudomonas aeruginosa* in mice and rat, *Edwardsiella ictaluri*, *Aeromonas hydrophila* and *Aeromonas veronii* in catfish, New Zealand rabbit, fish and mice were translated to colony forming units. LC_50_ of in vitro cell cultures of hepatitis A virus and Foot and Mouth Disease virus were translated to LC_1_, whereas effective dose fifty (ED-_50_) for Newcastle Disease vaccines was translated to ED_1 -_in chickens [5–20]. The method of Reed and Muench [21] as modified by Saganuwan [9] was used for LC_50_ determination in various laboratories. Protection index (PI) is equal to titration index = Nlog_10_ LD_50_, whereas N is number of titration using vaccine. In vivo LD_50_ value can be replaced by tissue culture LD_50_ (TCL_50_).

#### Derivation of LD_50_ formula

i. Modified formula of Reed and Muench
  ${\text{LD}}_{ 50} = \frac{MLD + MSD}{2}$ whereas MLD = Median lethal dose; MSD = median survival dose [9].

#### Derivation of LC_50_ formula

Conc. = initial concentration of colony forming unit per ml of sample = x

When concentration is double fold, triple fold and tetra fold, they are represented as 2 x X, 3 x X and 4 x X, respectively.

ii. Hence, $${\text{LC}}_{ 50} = \frac{x + 2x + 3x + 4x}{10} x 5$$
  $${\text{LC}}_{ 50} = \frac{10x}{10} x 5$$
iii. $${\text{LC}}_{ 50} = {\text{X}} \times 5$$
  x = initial concentration = colony forming unit; whereas LC_50_ = median lethal concentration that can kill 50% of test animals; x = initial concentration; multiplication factors for initial concentration = 10
iv. $${\text{x}} = \frac{{LC_{50} }}{5}$$
v. Number of colony forming unit (NCFU) per unit of sample [22]
  $${\text{NCFU}} = {\text{Nc}} \times {\text{Df}} .$$
  Nc = Number of colonies; Df = Dilution factor of the plate counted
vi. Therefore $${\text{CFU}} = \frac{Nc x Df}{N}$$
  Substitute x for CFU in equation v
  ∴$$\frac{{LC_{50} }}{5} = \frac{Nc x Df}{N}$$
  LC_50_ × N = 5(Nc × Df)
vii. $${\text{LC}}_{ 50} = \frac{{5\left( {Nc x Df} \right)}}{N}$$
viii. Median bactericidal concentration (MC_50_) formula is determined as follows
  ${\text{N}}_{\text{c}} = \frac{{N_{0} }}{{1 + e^{{r\left( {x - {\text{BC}}_{50} } \right)}} }}$ whereas N = Number of colonies for each plate.
ix. $${\text{BC}}_{ 50} = \frac{No}{2}$$
  Thus 2BC_50_ could replace MBC
x. ${\text{BC}}_{ 1} {\text{ = BC}}_{ 50} + \left[ {\frac{{l_{c} \left( {No - 1} \right)}}{r}} \right]$ whereas r = tangent slope on inflexion
  No could estimate the bactericidal intensity [23]
xi. Since the rate of bacterial load depends on the concentration of neutrophils. Exponent = (− kp + g)t, where k is the second-order rate constant for bacterial killing, p = neutrophil concentration; g = first-order rate constant for bacterial growth; t = time.
  K = 2 × 10^−8^ ml per neutrophil per min; g = 8 × 10^−3^ min
xii. When ${\text{P}} > \frac{g}{k}$ = critical neutrophil concentration

The critical neutrophil concentration = 3–4 × 10^5^ per ml, a value of ≤ 5 × 10^5^ predisposes human to bacterial infection [24]. All of the above formulas could be applied in determination of lethal concentration of immunogenic and anti-immunogenic agents in various models of vaccine development.

## Results

The colony forming unit, LC_1-_, median lethal concentration for each pathogenic microorganism, antigen, vaccine, animal model and their routes of administrations are presented in Table 1. The most virulent microorganism is Sheep Pox virus with LC_50_ value of 1.93 × 10^10^ cfu/ml followed by *Edwardsiella ictaluri* (2.8 × 10^4^ cfu/ml), Streptococcus pneumonia(10^4^–10^7^ cfu/ml) and Staphylococcus being the least virulent in rat with IC_50_ of 1.75 × 10^10^ cfu/ml, using intradermal, intraperitoneal, intravenous and intraperitoneal route of administration, respectively. Sheep was most susceptible, followed by catfish, mice and rat being the least susceptible in the present study (Table 1).

**Table 1:** The estimated colony forming unit and  median lethal concentration (LD_50_) of pathogenic microorganisms’ antigens and vaccines

Pathogenic microbes	Animal model	Antigen(s)/Strain	Route	CFU (LC_1_)	LC_50_ cfu/ml	Comments	Reference(s)
*Staphylococcus aureus*	Mice	pC1-Neo pC1-Neo-MeccA	Intraperitoneal	2.2 × 10^7^	1.1 × 10^8^	Less virulent	[5]
*Staphylococcus aureus*	Mice	Fibrinogen Fibronectin	Intravenous	2 × 10^6^	1 × 10^7^*	Less virulent	[6]
*Staphylococcus aureus*	Mice	Endotoxin-free PBS	Intraperitoneal	1 × 10^8^	5 × 10^8^*	Less virulent	[10]
New Castle disease virus	Chicken	Lasota vaccine	Oral	0.84–1.92	4.2–9.6/ml	Very virulent	[11]
New Castle disease virus	Chicken	12 vaccine	Oral	1.14–1.92	5.7–9.6/ml	Very virulent	[11]
Hepatitis A Virus	In vitro	HAV AM75/18F	In vitro	2.8 × 10^6^	1.4 × 10^7^	Less virulent	[12]
*Streptococcus pneumoniae*	Mice	Pneumococcal surface protein A	Subcut	9 × 10^4^–10^6^	4.5 × 10^4^–10^6^	Moderately virulent	[13]
*Streptococcus pneumoniae*	Mice	PSPA1 and 2 bound to Vi polysaccharide	Intravenous	2 × 10^3^–2 × 10^6^	10^4^–10^7^	Moderately virulent	[14]
Foot and Mouth Disease virus	In vitro cell line (hamster kidney 21 cell line)	Serotype A, 0 and SAT-2	Cell culture	2.8 × 10^8^	1.4 × 10^9^	Less virulent	[15]
*Staphylococcus aureus*	Rat	Strain (ATCC29213)	Intraperitoneal	3.5 × 10^9^	1.75 × 10^10^	Less virulent	[16]
*Pseudomonas aeruginosa*	Rat	(ATCC27853) strain	Intraperitoneal	6 × 10^7^	3.0 × 10^8^	Less virulent	[16]
Sheep pox virus	Sheep	SPPV strain (Hd 2012)	Intradermal	3.86 × 10^2^	1.93 × 10^3^	Highly virulent	[17]
*Edwardsiella ictaluri*	Catfish	Suspension of *E. ictaluri*	Intraperitoneal	5.6 × 10^3^	2.8 × 10^4^	Moderately virulent	[18]
*Aeromonas hydrophila*	New Zealand rabbit, fish	Glycoprotein based-vaccine	Intradermal	1 × 10^9^	5 × 10^9^	Less virulent	[19]
*Aeromonas veronii*	Fish mice	Bacteriovorax strain H_2_	Oral	7.2 × 10^8^	> 10^9^ PFUg^−1^	Less virulent	[20]

*CFU* colony forming unit

* = sublethal dose; highly virulent = statistically significant in relation to CFU/viral concentration; moderately virulent-statistically significant in relation to CFU/viral concentrations; Less virulent = statistically not significant in relation to CFU/viral concentrations

## Discussion

The median lethal concentration (1.1 × 10^8^ CFU) for plasmid cloned neomycin (PC1 = Neo) and plasmid cloned neomycin methicillin resistance *Staphylococcus aureus* (PCl-Neo-MeccA) and 1 × 10^7^ CFU for *S. aureus* fibrinogen in mice show that the microorganism is less virulent [5]. However, endotoxin-free phosphate buffered-saline (PBS) did not show lethality at 5 × 10^8^ CFU [10]. The findings agree with the report indicating that active vaccination with a mixture of recombinant penicillin binding protein 2a in rabbit (rPBP2a/r) autolysin reduced mortality in methicillin resistant *Staphylococcus aureus* and protected mice against infection [7]. Higher level of autolysin specific antibodies has a predominant immune globulin G_1_ (lgG_1_) indicating that *S. aureus* is opsonized in serum of immunized mouse and could increase phagocytic killing [10]. But the lower concentration of New Castle Disease (NCD) Lasota (4.2–.6/ml) and 12 vaccine (5.7–9.6/ml) that offered protection against New Castle Disease may suggest robustness of the vaccines as compared to effective dose 50 (ED_50_) of B1 strain (5.1–20.9/ml), C30 strain (1.1–22/ml) and Villegas-Glisson University of Georgia (VG-VA) strain (0.3–16.2/ml), respectively [11]. But pneumococcal surface protein A (PspA^3+2^) is better than PspA^2+4^ and PspA^2+5^ vaccine in respect of cross protection against pneumococcal infection [13]. The conjugated α helical region of PspA to Vi enhanced protective immune response and provided protection against pneumococcal infection [14]. Antibody elicited by PspA recombinant protein and DNA vaccine proffer humoral response which is different from fragment crystallizable (Fc), (lgG1/lgG22 ratios) and fragment antigen-binding (Fab) epitopes of the induced antibodies [22]. The tissue culture lethal dose 50 (TCLD_50_) determined by Cormier and Janes showed that zeolite could be used against hepatitis A virus infection [12]. Foot and mouth disease (FMD) titer of serotype A, O and SAT-2 from the roller cultivation system provided protection at 2 weeks post-vaccination [15]. The LC_50_ of *S. aureus* (1.75 × 10^10^ cfu/ml) and *P. aeruginosa* (3.0 × 10^8^ cfu/ml) show that the microorganisms are less virulent [16]. The pathogenicity is based on clinical signs, survivability and postmortem changes of the infected animal. Therefore, the LC_50_ of 1.93 × 10^3^ shows that the intradermal Romanian SPPV is a potent vaccine for control and prevention of sheep pox in a disease-free or endemic country [17]. *Edwardsiella ictaluri* is moderately pathogenic in *Pangasionodon hypophthalamus* with LC_50_ of 2.8 × 10^4^ cfu/ml and caused necrosis of liver and haemolysis [18]. Vaccination against *A. hydrophila* using glycoproteins (5 × 10^9^ cfu/ml) with ginseng, provided reliable immunity in fish and rabbit [19], though the immunity may not be strong. Bacteriovorax strain H_2_ is relatively safe in mammalian bio system including snakehead and could be used as a probiotic agent for the bio control of *A. veronii* infection in snakehead [20]. As a number of promising protein-based and whole cell vaccines are currently undergoing different phases of development [29], microorganisms and antigens with lower LC_50_ values are more pathogenic and may require higher doses of vaccines. More so, different bacteria have different incubation periods and mixed infection decrease incubatory period and longevity of the host [22]. Pathogenicity is multifactorial with genetic regions associated with virulence and resistance determinants. Although pathogenicity islands (PAIs) and resistance islands (RIs) play great role in bacterial infection [25]. Pathogenicity Island (150-kb) encodes several genes for pathogenesis and antibiotic resistance [26]. Therefore pathogenicity is qualitative whereas virulence is quantitative [27]. Pathogenicity islands are acquired by horizontal gene transfer that promote genetic variability described as evolution quantum leaps involving large amounts of DNA [28]. Mechanisms of pathogenicity are via lysis of cell wall, toxin, adhesins and invasion of host cell [29]. Application of monitoring programs, prudent use of guidelines and campaigns could minimize the transmission and spread resistant bacteria [30, 31]. Pathogenic potential of microbes is a continuous phenomenon [32] that is related to infective dose and virulence [33]. Hence, host–pathogen parameters give progression of infection and may lead to survival or death [34]. But sometimes cell lines are used and the information related to intercellular mechanism is lacking [35], making it difficult to predict ideal pathogenicity/virulence, most especially in in vitro-in vivo translation. However, molecular basis of pathogens has made possible, identification of many therapeutic interventions [36], as evidenced by disease-gene-drug interaction [37], during the late stage of new antibiotic development. This can help pharmaceutical companies that have limited resources to discover and develop new antibiotics [38] for emerging and rare diseases that may need orphan drugs [39].

Determination of pathogenicity using a revised arithmetical method of Reed and Munch [9] is an application of computational biology, which is the science of using biology to develop algorithms or models for understanding biological relationship [40] that involves data analysis and interpretation [41]. Using heterogeneity of animal models in the present study and the data generated, pose a special challenge [42], which could be summarized by expanding the computation that would find a range of value, which would serve as basis for determination of one or more biological parameters [43]. In the present study, the LC_50_ of pathogenic microorganisms, antigens and titrated antibodies should be sought between 1.93 × 10^3^ and 1.75 × 10^10^ CFU/ml depending on the in vitro or in vivo test models, route of inoculation and pathogenicity of the test pathogen, antigen and titrated antibody [44]. Computational immunology may translate to the possibility of all mammals having homogeneity of immunogenes from evolution [45]. Data derived from complex processes driven by evolution [46], and deep learning methods as complicated by powerful programmed machine with improved software infrastructures, may not provide ultimate solution for the field of computational biology [47], making the present study very relevant.

Diversity of quasispecies predicts a limit between mutation rate, population dynamics and pathogenesis [48] via mathematical modeling, that may produce results similar to hypothetical and real experiments [49]. The locus that determines pathogenicity may be involved in lipopolysaccharide biosynthesis [50]. Also, pathogenicity of a microbe varies with the genetic background of mouse strain [32]. The strategies used by pathogenic bacteria to cause pathogenicity are via cell wall, toxins, adhesins, invasion, intracellular lifestyles, regulation of virulence factor, evolution of bacterial pathogen, antibacterial resistance, pathogen-innate immune system interaction and viability of complete genome sequences [29]. But the evolution of pathogenicity is based on traits that ensure survival of microorganisms in their habitats [51]. Different pathogenic microbes isolated from host species have different incubation period. But when there is mixed infection, the incubation period decreases [22]. The pathogenicity index of 100µ per 10^6^ cfu may be applied for screening of *P. multocida* [52]. Influenza virus can affect colonization of *S. pneumoniae*, *S. aureus*, *N. meningitidis*, *M. tuberculosis*, and *S. pyogenes*, RSV, Rhinovirus and HPIV. This has been proven by various mathematical models of microbial pathogenicity [53].

## Limitations

- The study was based on data generated in various laboratories; hence standard operating procedure (SOP) and general lab practice (GLP) may affect our findings.
- Differences in formulas may also affect the data generated.
- Routes of administration, animal models and variation in pathogenic molecules may affect the data generated.

## Data Availability

All data generated or analyzed during this study are included in this published article.

## Abbreviations

LC_50_: Median lethal concentration
LD_50_: Median lethal dose
BC_50_: Median bacterial concentration
N: Number of vaccine dose
T_1_: Titre index
LC_10_: Lethal concentration 10
x: Initial concentration
MLD: Median lethal dose
MSD: Median survival dose
NCFU: Number of colonies forming unit
Nc: Number of colony
Df: Dilution factor
e: Exponent
r: Tangent slope on inflexion
k: Second order rate constant
p: Neutrophil concentrations
g: First order constant for bacterial growth
t: Time taken to grow
SPPV: Sheep Pox virus
HAV: Hepatitis A virus
PBS: phosphate buffered-saline
pCl-neo: Plasmid cloned neomycin
pCl-neo-Mecca: Plasmid cloned neomycin methicillin *Staphylococcus aureus*
IgG_1_: Immunoglobulin G_1_
rPBP2a/r: Recombinant penicillin binding protein 2a in rabbit
NCD: New Castle disease
ED_50_: Effective dose 50
TCLD_50_: Tissue culture median lethal dose
PSPA: Pneumococcal surface protein A
Fc: Fragment crystallizable
Fab: Fragment anti-gen binding
VG-VA: Villegas-Glisson, University of Georgia
FMD: Foot-and-Mouth Disease
